# The National Cancer Institute Thyroid fine needle aspiration state of the science conference: a summation

**DOI:** 10.1186/1742-6413-5-6

**Published:** 2008-04-07

**Authors:** Zubair W Baloch, Edmund S Cibas, Douglas P Clark, Lester J Layfield, Britt-Marie Ljung, Martha Bishop Pitman, Andrea Abati

**Affiliations:** 1 Department of Pathology, University of Pennsylvania Medical Center. Philadelphia, Pennsylvania, USA; 2 Department of Pathology, Brigham and Women's Hospital and Harvard Medical School, Boston, Massachusetts, USA; 3 Department of Pathology, The Johns Hopkins Medical Institutions, Baltimore, Maryland, USA; 4 Department of Pathology, University of Utah Hospital and Clinics, Salt Lake City, Utah, USA; 5 Department of Pathology, University of California San Francisco, San Francisco, California, USA; 6 Department of Pathology, Massachusetts General Hospital, Harvard Medical School, Boston, Massachusetts, USA; 7 Laboratory of Pathology, National Cancer Institute, National Institutes of Health, Bethesda, Maryland, USA

## Introduction

On October 22 and 23, 2007, the National Cancer Institute (NCI) hosted "The NCI Thyroid Fine Needle Aspiration (FNA) State of the Science Conference," a two-day gathering in Bethesda, Maryland. Its mission was to establish a comprehensive interdisciplinary informational dialogue dedicated to thyroid FNA. Preparations for the conference began 18 months earlier with the designation of a steering committee, the appointment of nine working committees, coordination with co-sponsoring organizations, and the establishment of a dedicated permanent web site. The function of the website was to serve as a permanent educational tool for health care practitioners and patients alike, as well as to foster on-line dialogue.

Six committees were charged with preparing summary documents based on literature reviews on the following subjects: 1. Indications/Pre-FNA requirements; 2. Training and Credentialing; 3. Technique; 4. Terminology and Morphologic Criteria; 5. Ancillary Studies; and 6. Post-FNA Options for Testing and Treatment. Three additional committees were formed, one each to oversee the website, publications ensuing from the conference and the establishment of an on-line educational cytopathology atlas.

Literature reviews were limited to English language publications dating back to 1995, using PubMed as the search engine, with key words determined by the committee members. The first draft of the committees' summary documents ("Review and Conclusions") was posted on the web site and open for on-line forum discussion from May 1-June 30, 2007. There were several subsequent drafts and on-line discussion periods (Aug 15 – Sept. 30, 2007 and Nov. 30-Dec. 15, 2007). The documents underwent revision after each comment period prior to re-posting on the web. The two-day "live" conference in October, attended by 154 registrants, comprised of pathologists, endocrinologists, surgeons, and radiologists, gave the committees an in-depth opportunity to present their conclusions and debate controversial areas.

This is an abridged version of the topics reviewed at the conference and the website. This is not a "standards of practice" guideline, nor is it endorsed as such by the National Cancer Institute.

## I. Indications for Thyroid FNA and Pre-FNA Requirements

### A. Indications for performing an FNA of a thyroid nodule discovered by palpation

Every patient with a palpable thyroid nodule is a candidate for fine needle aspiration (FNA) and should undergo further evaluation to determine if an FNA is warranted[1]Thyroid nodules detected by palpation are usually at least 1.0 cm in dimension and are therefore clinically significant. Before a decision is made to perform an FNA, a complete history should be taken; a physical examination directed to the thyroid gland and cervical lymph nodes should be performed; and a serum thyrotropin level (TSH) and thyroid ultrasound (US) should be obtained [1,2].

Patients with a normal or elevated serum TSH level should proceed to a thyroid US to determine if an FNA needs to be performed (see section B below); those with a depressed serum TSH should have a radionuclide thyroid scan, the results of which should be correlated with the sonographic findings [1,2]. Functioning thyroid nodules in the absence of significant clinical findings do not require an FNA because the incidence of malignancy is exceedingly low [3]. A nodule that appears either iso- or hypo-functioning on radionuclide scan, however, should be considered for FNA based on US findings (see section B below) [1].

### B. Indications for performing an FNA of a thyroid nodule discovered via imaging

A nodule not previously suspected or discovered clinically, but detected by an imaging study, is considered an incidental nodule ("incidentaloma"). Incidentalomas detected by ^18^FDG-PET are unusual (2–3% of all PET scans) but have a higher risk of cancer (14–50%) compared to background incidence [4]. Therefore, a focal nodule that is ^18^FDG-PET-avid is an indication for FNA. This applies only to focal lesions. Diffuse increased uptake on ^18^FDG-PET does not warrant FNA unless thyroid sonography detects a discrete nodule.

All focal hot nodules detected on sestamibi scans and confirmed by US to be a discrete nodule should undergo FNA. Thyroid incidentalomas detected on sestamibi scans have a higher risk of cancer (22–66%) compared to baseline risk [5,6].

Incidentalomas detected by US (e.g., carotid Doppler scans or scans done for parathyroid disease) have a cancer risk of approximately 10–15% (0–29%) and should undergo dedicated thyroid sonographic evaluation [7-12]. Lesions with a maximum diameter greater than 1.0–1.5 cm should be considered for biopsy unless they are simple cysts or septated cysts with no solid elements. FNA may also occasionally be replaced by periodic follow-up for nodules of borderline size (between 1.0 and 1.5 cm in maximum diameter) if they have sonographic features that are strongly associated with benign cytology.

A nodule of any size with sonographically suspicious features can also be considered for FNA. Sonographically suspicious features include microcalcifications, hypoechoic solid nodules, irregular/lobulated margins, intra-nodular vascularity, and nodal metastases (or signs of extracapsular spread). This latter recommendation is controversial because it includes patients with microcarcinomas, in whom a survival benefit following an FNA diagnosis has not been documented. Nevertheless, the American Thyroid Association, the Academy of Clinical Thyroidologists and a collaborative effort of the American Association of Clinical Endocrinologists and the Associazione Medici Endocrinologi make this recommendation [1,7,13].

There are few direct data on the cancer risk of thyroid incidentalomas detected by computed tomography (CT) or magnetic resonance imaging (MRI). They are seen in at least 16% of patients evaluated by neck CT or MRI [14]. The risk of cancer in one study was predicted at 10%, but it included only a limited number of patients who went on to FNA[15]. CT and MRI features can not determine the risk of malignancy, except in very advanced cases that are unlikely to be incidental. Until more data are available, incidentalomas seen on CT or MRI should undergo dedicated thyroid sonographic evaluation. Any nodule with sonographically suspicious features (see above) should be considered for FNA. In addition, lesions that have a maximum diameter greater than 1.0–1.5 cm should also be considered for FNA (see above).

### C. Indications for performing a thyroid FNA using palpation vs. ultrasound for guidance

In the evaluation of individual patients with nodular disease, there are occasions when either palpation or ultrasound-guided (US-guided) FNA of a thyroid nodule are reasonable to perform. Palpation-guided FNA can be performed with high levels of success in specific circumstances [16-18]. The benefits of palpation-guided FNA of thyroid nodules are its reduced cost in comparison to US-guided FNA as well as its logistical efficiency: the practitioner can perform the procedure without an US machine or assistance from other practitioners.

Published data from one study concludes that US evaluation changes the management of 63% of patients with palpable thyroid nodules [19]. US findings like irregular margins, microcalcifications, intra-nodular vascularity and the characteristics of other occult thyroid nodules can be used to identify nodules at risk that should be sampled [12,20]. In several studies US guidance was shown to reduce the rates of non-diagnostic (i.e., insufficient cells and/or colloid) and false-negative aspirates [16,17,21]. Re-evaluation of patients using US-guided FNA for those with initially benign results on palpation-guided FNA, led to the reclassification of patients and the diagnosis of 14% more cancers in one series [22]. Finally, there are US-specific findings that can be used to inform the results of the US-guided FNA (e.g. the benign sonographic appearance of a unilocular cyst explains why only cyst fluid was obtained by FNA).

A palpation-guided FNA can be considered when a thyroid nodule >1 cm in maximum diameter has been confirmed via US examination of the thyroid. The sonographic examination is important, because physical examination can be imprecise in determining nodule size, its origin from the thyroid rather than adjacent tissues, and the degree of cystic change. US-guided FNA is acceptable and is preferred for nodules that are not palpable, >25% cystic or have been biopsied before and yielded a non-diagnostic result [23,24].

### D. The informed consent form for thyroid FNA

Legislation regulating the conditions under which consent must be obtained vary greatly by state [25-27]. Thus, providers (e.g., pathologists, radiologists, surgeons, endocrinologists, etc.) who perform thyroid FNA need to design informed consent policies and forms based on state regulations. For thyroid FNA, informed consent materials, including written documents, if used, should describe the FNA procedure and potential risks and complications. The possibility of the most frequently occurring complication, namely, hematoma, should be mentioned. Information should be presented in a manner to facilitate patient understanding. It might be useful to mention the possibility of a non-contributory result. Estimates of accuracy, such as false-negative and false-positive proportions, are not mandatory and should be considered only if the practitioner believes they would facilitate patient comprehension.

### E. Information required on the requisition form that accompanies a thyroid FNA

Federal regulations in the United States require that specific identifying information be provided to laboratories with all specimens submitted for laboratory testing [28]. These include the name and address of the person requesting the test; the patient's name or unique identifier; the patient's gender; the patient's age or date of birth; the name of test to be performed; the specimen source; the date of specimen collection; and any additional relevant information. The purpose of this discussion is to consider what 'additional relevant information' a laboratory needs to properly evaluate a thyroid FNA specimen.

The location of the nodule (right vs. left; isthmus; upper pole, mid-pole, lower pole, etc.) should be specified on the requisition form to permit correlation with sonographic findings and subsequent histopathologic examination (if any). Such identification is often necessary because patients often present with multiple nodules (some but not all of which may be biopsied), or they may develop other nodules over time.

There is, at best, an imperfect correlation between the size of a nodule and the likelihood of malignancy, but larger nodules (>4 cm) may be associated with a higher malignancy risk, and therefore size should be included [29].

Benign cytologic changes that mimic malignancy, particularly papillary carcinoma, occur in some patients with autoimmune (Hashimoto's) thyroiditis. If not alerted to this history, a misdiagnosis can occur. Furthermore, nuclear alterations may be seen in patients with a history of I-131 therapy (for hyperthyroidism) or external radiation [30]. In some patients with Graves' disease, an FNA of a nodule may include pleomorphic cells from the extra-nodular Graves' thyroid parenchyma that can be a pitfall in cytologic interpretation [31].

It is important to note a personal history of malignancy because metastatic tumors to the thyroid can mimic the appearance of a primary thyroid neoplasm. Metastatic renal cell carcinoma mimics a follicular neoplasm; melanoma can mimic medullary carcinoma; metastatic lung cancer can mimic anaplastic carcinoma of the thyroid. Cytologists should be alerted to the possibility of a metastatic tumor in any patient with a history of malignancy.

Approximately 15% of medullary thyroid cancers are familial (familial MTC or MEN2a or 2b). Knowledge of family history can alert the pathologist to the possibility of medullary carcinoma. Recent data show that papillary thyroid cancer can also be familial, and thus knowledge of such family history can alert the pathologist to consider papillary carcinoma.

Therefore, at a minimum, the following data should appear on the requisition form that accompanies a thyroid FNA to the laboratory:

1. Usual required data for lab test submission (see above)

2. Location of the nodule

3. Estimated size of the nodule

4. History of hypothyroidism, autoimmune thyroiditis, or a positive test for anti-thyroid antibodies

5. History of Graves' disease

6. History of I^131 ^or external radiation therapy, accompanied by the dates of treatment

7. Personal history of cancer

8. Family history of thyroid cancer

The following information can be useful to the cytologist but is considered optional on the requisition form: 1. additional clinical history, e.g., that a prior FNA was done, or that the patient is undergoing levothyroxine therapy. Morphologic alterations due to a prior FNA can affect cytologic interpretation, and levothyroxine use can alter follicular cell morphology. 2. TSH level. If a patient has Hashimoto's hypothyroidism or Graves' disease, cytologic findings can be affected. A lower serum TSH level is also associated with a lower risk of thyroid cancer. 3. results of US examination. 4. results of nuclear medicine imaging studies[32,33].

## II. Training and Credentialing for the Performance of a Thyroid FNA

### A. Training for the Performance of Thyroid FNA

Diagnostic accuracy of thyroid FNA is highly variable [18,34-38]. The majority of diagnostic failures are due to non-diagnostic samples or pathologists issuing diagnosis on samples with inadequate material [39]. Efforts toward improvement in proficiency in FNA sampling and specimen preparation should be given high priority. Reports comparing the effectiveness of specific, defined training strategies for FNA procurement are lacking and there is little agreement on what the definition of adequate training is. Merely performing a large number of FNAs does not improve results when controlled for level of training[40]. There is, however, evidence that when FNA specimen procurement is concentrated in fewer hands and when the same physician both procures and microscopically examines the specimen, the results improve [41,42]. Training in procurement has significant impact on results regardless of the method of guiding the needle (ie palpation vs. ultrasound guidance). The consistent and timely feedback on specimen quality provided to physicians who both procure and examine specimens microscopically helps to improve results as well. Ultrasound (US) guidance of the sampling provides evidence that the needle is correctly placed within the target.

Two reviews of FNA state that the procurement of the samples is not as easy as generally perceived and stress the importance of obtaining an adequate sample in order for the test to be useful [41,43]. Suen makes the recommendation that "the procedure is carried out by a core group of dedicated physicians" [44]. In a 2003 editorial Kocjan discussed many of the problems associated with the current practice of FNA and stressed the importance of training in specimen collection and preparation regardless of the specialty of the operator[45].

A recent publication on the teaching of procedural skills indicates that the most important factor for mastering procedures is focused training with appropriate feedback by expert practitioners [46]. The previously widely held belief that simply performing a large number of procedures produces excellence does not appear to be true [47]. Given these observations it doesn't make sense to advocate that a specific number of procedures should be performed without specifying the circumstances. Reports comparing the effectiveness of specific, defined training strategies for FNA sampling are lacking in the literature. Such studies would be helpful in order to optimize training of operators in various settings and specialties.

In addition to the harvesting of diagnostic material, training for technical excellence in sample preparation cannot be overemphasized. Several options are available for the preparation and processing of FNA specimens. On-site sample adequacy assessments are performed with smears which may be stained with a modified Giemsa or a rapid Papanicolaou technique. In order to optimize on-site adequacy smears, appropriate training in smear technique is imperative in any FNA training program regardless of specialty. On-site evaluation of the specimen adequacy has lessened the percent unsatisfactory specimens and limited the number of passes per nodule sampled [48-50]. In all but one of these reports on-site evaluation of the specimens was one of several factors reported to improve accuracy, but was not calculated as a separate factor [50]. One report found on-site evaluation helpful in minimizing unsatisfactory samples only for less experienced radiologists procuring samples [51]. At the very least, it appears that on-site evaluation serves as an important educational tool for the physician performing the FNA in providing immediate feedback on the quality of the specimen.

Training in US imaging technique and interpretation is beyond the scope of this review.

Components of FNA procurement training:

1. Studying of texts and DVD or similar teaching aids with moving images that explain the principles and show all required tasks including sampling and specimen preparation in detail. Lectures and demonstrations can also be helpful. A detailed instructional video done by Dr. Britt-Marie Ljung is available to all, sponsored by the Papanicolaou Society of Cytopathology on their website under the PSC Guidelines link.

2. Bench practice of sampling and smearing on bovine liver or similar safe material, under supervision. Timely and precise placement of the needle tip under ultrasound guidance can be practiced with a model (for example turkey breast with a "target" inserted between the muscles or commercially available practice materials).

3. Sampling of thyroid nodules guided either by palpation or US, supervised by a proficient operator, and followed by examination of all samples to provide timely feed back on quality of samples.

4. Number of cases needed for achieving proficiency will vary depending on individual background and aptitude as well as case mix. Although it is best to start with easier cases, challenging cases need to be included. One should expect at least 90% diagnostic specimens before training is complete. The sampling of thyroid cysts devoid of follicular cells designated as "cyst fluid only" and categorized as " non-diagnostic" should not be considered "non-diagnostic" for credentialing purposes.

### B. Credentialing and re-credentialing

Complete residency/fellowship training including FNA procurement or equivalent training in an alternative setting should suffice for initial credentialing.

For re-credentialing, documentation of the number of total FNA procedures per year, for an individual provider, in combination with a documented unsatisfactory sample rate (<10%) is a conservative measure of proficiency. The sampling of  thyroid cysts devoid of follicular cells designated as “cyst fluid only” and categorized as “ non-diagnostic” should not be considered “non-diagnostic” or “unsatisfactory” for credentialing purposes. 

## III. Techniques for the Performance of Thyroid FNA

### A. Aspiration devices, needles and methods

A wide variety of needles of varying lengths and diameters are available for FNA. Commonly available 27–22 g needles are best used for thyroid FNA with 25–27 g needles being preferred for the initial biopsies and increasing needle size if needed; larger diameter needles are reserved for drainage of viscous colloid cyst contents. A variety of syringe holders are also available; among the oldest and most widely used pistol grip-like holders is the Cameco syringe gun. Other aspiration devices include the Tao instrument and the Inrad aspiration biopsy syringe gun.

The native suction provided by surface tension within smaller diameter needles often make devices for additional suction unnecessary [52]. When suction is needed, such as in the drainage of cyst contents, the Zajdela technique can still be used by having a section of IV tubing interposed between the needle held by the physician and the aspiration device held by an assistant.

The basic principals of thyroid FNA are described in detail elsewhere and are the same whether the needle is inserted into the lesion using manual palpation or ultrasound guidance [53]. As mentioned previously, a detailed instructional video is available at the website of the Papanicolaou Society of Cytopathology. When visualized with ultrasound, different areas of large masses should be sampled. If the nodule is complex, the wall, solid elements and suspicious calcified areas should be sampled avoiding cystic areas. Cellular material is obtained by the cutting action of the trailing edge of the needle (heel of the bevel) and is retained in the needle core by forward motion and capillary tension. As a starting point, a dwell time of 2–5 seconds within the nodule with 3 forward and back oscillations per second usually maximizes cellular yield, minimizes bloody artifacts, and efficiently produces 1–2 slides per biopsy pass.

Easily learned and very effective smearing techniques allow the aspirated material to be presented on the slides for optimal fixation, staining, and microscopic assessment [53]. Direct smears are necessary for immediate assessment at the time of biopsy. The failure or any significant flaw in smearing technique can limit or totally hinder microscopic evaluation, irrespective of how much material is obtained during the biopsy. Poor smear training and other logistical issues with respect to specimen transport to the laboratory have led to the utilization of a secondary means of specimen preparation, liquid based cytology (LBC), to be used in some laboratories [see specimen processing below].

Currently, the ideal physician to perform thyroid FNA in an institutional or office practice should be experienced, having repeatedly demonstrated appropriate judgment in nodule selection, technical excellence, and proficiency in obtaining aspirate material and preparing slides. For ultrasound guidance of FNA for either non-palpable or palpable nodules, this physician must have high resolution ultrasound imaging equipment with high frequency linear array transducers available, appropriate diagnostic skills, and documented experience in performing ultrasound-guided FNA.

### B. Anesthesia

Most thyroid FNAs are well-tolerated and are not associated with significant patient discomfort or pain; however, the use of local anesthesia insures that the procedure will not be painful, and offers peace of mind, resulting in an overall more comfortable experience. For this reason, the trend among many experienced FNA physicians is to use local anesthesia for all thyroid FNAs[54]. However, local anesthetic may cause difficulty in subsequent sample evaluation. For deep, non-palpable thyroid nodules that may require more time and probing to reach the nodule, and for all biopsies using needles other than a fine needle, local anesthesia is recommended.

The local anesthetic of choice is 1% lidocaine or Lidocaine 2% Epi l:100,000. This may be performed through the injection of about 0.5 cc of the anesthetic utilizing a long bevel injection needle, (not a TB 27 g injector), slowly into the subcutaneous fat (not the dermis) thus allowing the anesthetic to back infiltrate the dermal nerves rather than make a painful intradermal wheal.

### C. Core biopsy

There is limited literature regarding the use of modern biopsy needles, but so far it suggests that it is safe, well tolerated, and associated with a low incidence of complications. This is mainly due to the use of single action spring activated needles, i.e. Temno needle, which utilizes a non-advancing cutting action, or double action spring loaded needles (i.e. Monopty or Biopty gun) with a short throw device (11 mm excursion) [55,56].

FNA remains the best technique available to date for the initial evaluation of thyroid nodules. The slight increase in diagnostic accuracy obtained by CNB is outweighed by ease of use, cost effectiveness, and less patient discomfort associated with FNA [57]. Ultrasound-guided CNB should not be seen as a competitor of FNA, but rather as a complementary investigational tool. CNB under ultrasound guidance utilizing modern needles may be advantageous in cases rendered "unsatisfactory" by FNA, but offers no additional diagnostic value in separating a cellular hyperplastic nodule from follicular adenoma and follicular carcinoma [57] When cytopathology expertise is not available, CNB provides a small tissue sample for histologic examination.

### D. Preparation of FNA material for Routine Evaluation

Aspirated tissue or cyst fluid may be directly smeared for air-dried and alcohol fixed preparations for staining with Romanowsky (Diff-Quik, Wright-Geimsa, Wright stains) and Papanicolaou stains, respectively. Direct smears can be processed alone or with a supplemental LBC or cellblock prepared. Likewise, liquid based processing can be utilized either alone or as a supplement to direct smears. Direct smears, however, are essential for immediate assessment. For LBC, the aspiration needle should be flushed with a small amount (~0.5 cc) of liquid (Cytolyt™, balanced saline or Hank's solution) and placed in a Falcon tube for transport to the lab. For remote transport or for specimens expected to have delayed processing, a fixative such as Preservcyt™ or CytoRich Red™ is necessary for optimal cellular preservation. This cell-rich liquid can also be used for cellblock preparation if needed. Residual cyst fluid may be submitted to the lab fresh or fixed for further processing, either by LBC or cellblock.

Immediate assessment is widely utilized. Some laboratories employ it routinely for all thyroid FNAs as it may decrease complications, improve triage of tissue and determines sample adequacy [23,48,51,58-62]. One study found that immediate assessment as currently practiced, increases adequacy by up to 20%, but places a significant cost and burden on the laboratory [63].

### E. Optimal number of passes

It is not possible to define a specific number of passes that should be used in every setting. Although studies show that the more passes performed the higher the adequacy, between 2 and 5 passes are a reasonable number of passes to perform to try and ensure an adequate sample [50,63,64]. There is no justification to recommend a different number of passes for a cystic lesion, unless the criteria for adequacy are different. A reasonable protocol is as follows: FNAs with rapid interpretation available: 2 biopsies from different areas of the lesion with a representative slide stained for adequacy. No more tissue is needed if a cyst is completely drained and no residual mass is identified, a specific malignancy is identified (and no ancillary tests are deemed necessary), or if the aspirate appears adequate. Additional biopsies are recommended if there is a residual mass after draining a cyst, cellularity is inadequate or to enrich a sample for cellblock or ancillary studies.

For FNAs without a rapid interpretation available: 2–5 biopsies from different sites with representative tissue from each pass smeared on a slide (or 2) and/or the tissue rinsed into a collection tube for either LBC or cellblock preparation.

### F. Adequacy of Solid and Cystic lesions

Given that the purpose of thyroid FNA is to provide clinically useful information regarding the need for surgery, the FNA sample must be adequate enough for an interpretation that yields a low false negative rate. A thyroid FNA that is persistently inadequate may or may not result in surgery, depending on the clinical and ultrasounographic considerations [23,65].

Adequacy defines the quality and quantity of a sample, a definition that varies not only with respect to the site sampled, but also with respect to the type of lesion sampled. The cellularity of a specimen is influenced by the intrinsic nature of the lesion, and, as such, the same definition of adequacy does not apply to all specimen types [66].

All thyroid FNAs, however, must be technically adequate with well-preserved and well-prepared tissue for interpretation. Any cytological atypia precludes the interpretation of an inadequate sample and, although adequacy may be deemed "limited", an interpretation of "atypical" must be rendered, preferably with an explanation of the atypia.

An interpretation of an inflammatory process such as thyroiditis does not require a minimum number of follicle cells. An interpretation of a colloid nodule in which there is abundant, thick colloid present does not require a minimum number of follicle cells [66-68]. In solid nodules producing a follicular cell population with less than abundant colloid, a minimum number of 5–6 groups with a least 10 cells is recommended, preferably on a single slide [50,69,70]. Thyroid cysts with little to no follicular cells should be interpreted as "cyst fluid only" under the heading "nondiagnostic" and not "unsatisfactory". An optional recommendation for correlation with the cyst size, complexity, and ultrasonographic character may be added.

## IV: Diagnostic terminology/classification scheme and morphologic criteria for cytologic diagnosis of thyroid lesions

### A. Diagnostic terminology/classification scheme for thyroid fine-needle aspiration (FNA) interpretation

Several classification schemes have been suggested by various authors based on personal/institutional experiences and clinical organizations [9,13,66,71-74]. A recent survey of pathologist and clinicians on the perceptions of diagnostic terminology and cytopathology reporting of thyroid FNA, showed a discord between pathologists and clinicians[75].

Many studies favor a tiered system for classifying thyroid FNA; this ranges from 3–6 (or more) diagnostic category schemes. The most favored one is a six category diagnostic scheme consisting of benign, lesion (atypia) of undetermined significance, follicular neoplasm, suspicious, malignant, and unsatisfactory [71-75]. A suggested scheme is as follows (summarized in Table 1):

**Table 1:** Accepted Thyroid FNA Classification Schemes

**Suggested Categories**	Alternate Category (s) terms*	Risk of Malignancy**
Benign		<1%
Follicular lesion of undetermined significance	Atypia of undetermined significance	5–10%
	R/O Neoplasm	
	Atypical follicular lesion	
	Cellular Follicular Lesion	
Follicular Neoplasm	Suspicious for follicular neoplasm	20–30%
Suspicious for Malignancy		50–75%
Malignant		100%
Non-diagnostic	Unsatisfactory	

*These terms can be used instead of the suggested category terms (based on website responses and NCI meeting attendees); ** Data collected from literature [71-73, 80, 98]

1. Benign

a. Low risk of malignancy

b. The diagnostic terms in this category include but are not limited to nodular goiter, chronic lymphocytic thyroiditis, hyperplastic/adenomatoid nodule in goiter and colloid nodule.

c. Patients with a benign nodule are followed by clinical and periodic radiologic examination and some patients may undergo repeat FNA due to increase in the size of nodule.

2. Follicular Lesion of Undetermined Significance/Atypia of Undetermined Significance

a. Risk of malignancy 5–10%.

b. This is a heterogeneous category that includes cases in which the cytologic findings are not convincingly benign, yet the degree of cellular or architectural atypia is not sufficient for an interpretation of "Follicular Neoplasm" or "Suspicious for Malignancy".

c. Some cases are placed in this category because of a compromised specimen (e.g. low cellularity, poor fixation, obscuring blood).

d. This group can benefit from repeat FNA and correlation with clinical and radiologic findings.

e. This category is optional and when utilized should ideally represent less than 7% of all thyroid FNA interpretations.

3. Follicular-Neoplasm/Suspicious for Follicular Neoplasm

a. Low to intermediate risk of malignancy 20–30%.

b. This category applies to ***non-papillary ***follicular patterned lesions/neoplasms and Hurthle cell lesions/neoplasms. A majority of studies have shown that up to 20% of the thyroid lesions classified as such are found to be malignant on surgical excision (the predictive value/relative risk of this diagnosis can be included in the report). This percentage may be higher in Hurthle cell lesions if the nodule is equal to or larger than 3.5 cm in greatest dimension.

c. Other suggested diagnostic terms for this category are micro-follicular proliferation/lesion, suggestive of neoplasm and follicular lesion.

d. Most patients with this diagnosis will undergo lobectomy/hemithyroidectomy and a definite diagnosis (adenomatoid nodule vs. adenoma vs. carcinoma) is rendered on surgical pathology examination.

e. Some laboratories prefer the term "Suspicious for Follicular Neoplasm" may also be used for its clarity and for risk-management reasons.

4. Suspicious for Malignancy

a. This term can be used as:

i. Suspicious for papillary carcinoma (a majority of cases in this group (50–75%) are found to be follicular variant of papillary carcinoma).

ii. Suspicious for medullary carcinoma (applies to cases in which there is limited specimen to perform confirmatory immunostains for calcitonin. The cytology report should include a recommendation to assay serum calcitonin levels to confirm cytologic impression).

iii. Suspicious for other primary and secondary malignancies

iv. Suspicious for neoplasm because of total necrosis of the lesional cells (anaplastic carcinoma).

5. Malignant

6. Non-diagnostic

a. Specimen processed and examined, but non-diagnostic due to limited cellularity, no follicular cells or poor fixation and preservation. A repeat FNA can be recommended in these cases (see discussion on Post-FNA Testing and Treatment Options).

### B. Morphologic Criteria

#### 1. Morphologic criteria of benign, non-neoplastic conditions

**Nodular Goiter: **The cytology specimen from a goiterous nodule (depending upon the preparation method) is characterized by:

• Abundant watery colloid (usually appears bluish-pink magenta in color and shows a "chicken wire" artifact due to air-drying in smears stained with Romanowsky stain).

• Follicular cells appear small, round to oval in shape with dark nuclei and are arranged in monolayer sheets, groups with follicle formation or as single cells[76,77]. Macrophages, usually filled with hemosiderin granules are also noted; however, their number depends upon the presence or absence of degenerative changes or a cystic component [77].

##### Autoimmune Thyroiditis/Chronic Lymphocytic Thyroiditis/Hashimoto's Thyroiditis

The FNA is usually performed in patients with thyroiditis who present with distinct nodules that are cold on thyroid scan. [78]The specimens from such cases usually show:

• Scant colloid, Hürthle cells, follicular cells, lymphocytes and few plasma cells.

• The lymphocytes are usually seen in the background, percolating between cell groups and in some cases one may see an intact lymphoid follicle. The Hürthle cells may display nuclear atypia and similarly follicular cells may show some chromatin clearing and nuclear grooves; however, one should refrain from interpreting these changes as malignant [78].

• An extensive lymphocytic infiltrate can appear monotonous and mistaken for malignant lymphoma arising in lymphocytic thyroiditis [78,79]. If one suspects lymphoma it is advisable that an aliquot of specimen be submitted for flow cytometry to confirm the morphologic suspicion.

#### 2. Morphologic criteria of follicular-patterned lesions

• Fine-needle aspiration (FNA) is a screening test for follicular patterned lesions of the thyroid because it cannot distinguish between benign and malignant non-papillary follicular and Hurthle cell lesions. The cytopathologists often struggle with the diagnosis of follicular patterned lesions due to the lack of well-defined cytomorphologic criteria (both benign and malignant lesions appear similar in cytologic specimens) and standardized terminology [80,81]. This is further complicated by the lack of "meeting of minds" in the histologic diagnosis of these lesions [82]. To rely on cytologic atypia in follicular and Hürthle cells to differentiate between benign and malignant lesions has not proven to be a reproducible criterion [83]. A number of benign conditions such as thyroiditis, post treatment effects and adenomatoid nodules can show marked cellular atypia [78,79].

##### Follicular lesion of undetermined significance/atypia of undetermined significance

FNA specimens in this category include cases that partly display features of both hyperplastic/adenomatoid nodules and follicular neoplasm i.e. cellular specimen showing follicular cells arranged in cohesive groups and microfollicles with focal nuclear crowding and overlapping and monolayer sheets in a background of watery colloid admixed with thick colloid and few macrophages. The question arises as to how useful it may be to divide non-papillary follicular lesions into two separate categories; should all be diagnosed as indeterminate follicular lesions with the decision of how to manage being left to the clinician via either clinical follow-up and repeat FNA or surgical excision. Repeat FNA has been shown to play an important role in the management of thyroid nodules. Published experiences have shown that by repeating the FNA >50% of nodules which are non-diagnostic or indeterminate on initial cytologic diagnosis can latter be placed into definite diagnostic categories with repeat FNA [71,73].

##### Follicular neoplasm/Suspicious for follicular neoplasm

This term encompasses both benign and malignant tumors; i.e. follicular adenoma and carcinoma. The FNA of a follicular neoplasm usually shows:

• Hypercellularity as compared to most aspirates of nodular goiter demonstrating a monotonous population of follicular cells with minimal or absent background colloid. The cells are usually arranged in three dimensional groups and microfollicles with prominent nuclear overlapping and crowding. Some cases may show nuclear atypia; however, this is not a diagnostic criterion of malignancy, since benign nodules can also show nuclear atypia [84].

• The presence of microfollicles in an FNA specimens may be diagnostic of a follicular lesions neoplasm (adenoma or carcinoma) [85]. Some authors have proposed the term *micro-follicular lesion *[75]. However, various studies have shown that the interpretation of microfollicles suffers from inter-observer variability. Additionally, aspirates of normal thyroid and hyperplastic/adenomatoid nodules can show microfollicles [86]. Others have suggested that diagnosis of follicular neoplasm should only be used when a thyroid FNA specimen demonstrates a monotonous cell population arranged in cohesive groups with nuclear overlapping and crowding in a background if thick instead of watery colloid [80].

##### Hurthle cell neoplasm

FNA specimens of Hürthle cell lesions (benign and malignant) usually show:

• Cellular aspirate comprising a single cell population of Hürthle cell in a background of minimal colloid. Cells can be arranged in monolayer sheets, follicular groups or as scattered single cells [87]. Some authors have suggested that cellular dispersion leading to single cells is more common in aspirates of Hurthle cell carcinoma than adenoma; however, this observation has not been validated [87]. Cellular atypia is also commonly observed in Hurthle cell lesion; this can be seen in the form of random nuclear enlargement, multi-nucleation, cellular pleomorphism and prominent nucleoli.

• FNA specimens of neoplastic Hurthle cell lesions may show intra-cytoplasmic lumens and transgressing vessels [88].

##### Follicular Variant of Papillary Carcinoma

Similar to the histologic diagnosis the cytologic interpretation of FVPTC can also be difficult. The FNA specimens of FVPTC usually show:

• Tumor cells arranged in monolayer sheets and follicular groups in a background of thin colloid. Thick colloid can also be present, however, much less as compared to classic PTC. The tumor cells show nuclear elongation, chromatin clearing and thick nuclear membranes; however, nuclear grooves and inclusions are rare. Nonetheless, chromatin clearing and nuclear membrane thickening is always seen in these cases [89,90].

• If a thyroid FNA specimen focally shows cells with nuclear elongation, chromatin clearing and grooves but lacks nuclear inclusions, it may be diagnosed such as "follicular neoplasm with features suspicious for PTC.

#### 3. Morphologic Criteria of Malignant Tumors

The well-differentiated thyroid carcinomas are the commonest form of malignant thyroid tumors. They are more common in young adults, whereas, the less differentiated and anaplastic tumors of the thyroid are prevalent in older age.

##### Cytology of Papillary Thyroid Carcinoma (PTC) and its variants

The cytologic features of PTC can be divided into major and minor diagnostic features; these are as follows:

• **Major Diagnostic Criteria**: Enlarged, oval "and irregular" nucleus, eccentric and often multiple micro-nucleoli, fine, pale chromatin, longitudinal intranuclear grooves and intranuclear pseudo-inclusions [76,91].

• **Minor Diagnostic Criteria: **Papillary cyto-architecture, syncytial monolayers, dense squamoid cytoplasm, "Bubble gum" colloid, psammoma bodies, multinucleated giant cells, histiocytoid cells and cellular swirls [92-94].

**Follicular variant of papillary carcinoma**: Refer to section on follicular patterned lesions of thyroid.

##### Tall cell variant of papillary carcinoma

Cytology specimens of this tumor usually show elongated cells with sharp cytoplasmic borders, granular eosinophilic cytoplasm and variably sized nuclei with nuclear features of papillary carcinoma. The nuclear features of papillary carcinoma are usually abundant and readily identifiable as compared to other variant of PTC [95].

##### Medullary Thyroid Carcinoma

The FNA specimens from MTC can show varied morphologic pattern similar to that seen in surgical pathology specimens. The majority of MTC cases show:

• A cellular aspirate consisting of round to oval cells arranged mainly as single cells or loosely cohesive groups. The individual tumor cells show abundant eosinophilic granular cytoplasm and up to 20% of cells will demonstrate fine granules in Romanowsky-stained preparations. The nuclei are usually eccentric giving rise to a plasmacytoid appearance to tumor cells.

• The nuclear chromatin is similar to that seen in neuroendocrine tumors; salt and pepper type with inconspicuous nucleoli. Intranuclear inclusions and multinucleated cells can also be seen.

• In some cases of MTC the tumor cells are can assume a "spindle shape" and appear mesenchymal in origin. Amyloid may be observed as acellular material in the form of strings or as round to oval shaped fragments.

• In cytology specimens the diagnosis of MTC can be confirmed by performing immunostains for Calcitonin. In cases with limited cellularity it is advisable to have a serum Calcitonin levels performed on the patient to confirm the diagnosis of MTC [76,96].

##### Anaplastic carcinoma

The aspirates from anaplastic carcinoma usually do not pose any diagnostic difficulties; they can be readily classified as malignant due to extreme cellular pleomorphism and obvious malignant features [97,98].

## V. Utilization of Ancillary Studies in Thyroid FNA

### A. Indications for ancillary studies in thyroid FNA

Medullary carcinoma and anaplastic carcinoma are the most common primary thyroid tumors that may require additional immunohistochemical studies to confirm the diagnosis. Although rare, metastatic tumors may occur in the thyroid and a history of a known primary along with a pertinent immunohistochemical panel would be helpful in making a final diagnosis. The utilization of ancillary studies to re-classify an indeterminate or suspicious FNA into a benign or malignant category or to refine the risk of malignancy within this category is controversial.

### B. Specific ancillary studies to be performed for each of these indications

The ancillary studies with the widest utility involve the detection of specific proteins using immunologic techniques, typically immunohistochemistry on cell block preparations. Immunocytochemistry may also be utilized but only after careful validation of the protocols for this type of specimen. In cases of suspected medullary carcinoma, an immunohistochemical panel of calcitonin, thyroglobulin, CEA, and chromogranin can be confirmatory. Immunohistochemical findings should be correlated with serum calcitonin levels, particularly if there is insufficient FNA sample for ancillary studies [99-106]. Care should be employed in evaluating the results of calcitonin staining on cell block preparations of thyroid neoplasms with oncocytic features as cases of non-specific staining of oncocytic cells have been documented. Endogenous biotin in follicular cells may also cause non-specific staining.

Anaplastic carcinoma is often apparent based on its pleomorphic cytomorphology and aggressive clinical presentation. Anaplastic carcinoma often lacks TTF-1 and thyroglobulin staining. However, IHC for pan-cytokeratin may be utilized to distinguish anaplastic carcinoma from sarcomas. The clinical setting may also raise the possibility of a metastatic lesion [107-112]. The most common metastases to the thyroid arise from primary carcinomas of the kidney, lung, breast, colon, or malignant melanoma. The clinical history and presentation is important in determining the appropriate ancillary studies. One may initially employ TTF-1 and thyroglobulin IHC to narrow the primary site to thyroid, followed by further IHC characterization if these stains are negative [113].

One challenging area is excluding the possibility of lymphoma in the setting of Hashimoto's thyroiditis. All cases of Hashimoto's thyroiditis should not be automatically sent for flow cytometric immunophenotyping. In addition, immunophenotyping results from thyroid FNA samples should be interpreted with caution since Hashimoto's thyroiditis may yield κ/λ ratios that are skewed beyond normal values associated with reactive lymph nodes [114]. The indication for flow cytometric immunophenotyping should be based on cytomorphologic or clinical features that raise the suspicion of lymphoma. Parathyroid tissue can be extremely difficult to distinguish from thyroid tissue based on cytomorphologic features alone. In this setting, IHC for TTF-1, PTH, and chromogranin may be helpful. Neither the IHC nor the cytomorphology should be utilized to distinguish normal from abnormal parathyroid tissue. Chemical detection of PTH levels in FNA samples has been utilized, and may be considered following careful assay validation [115-120]. The identification of metastatic thyroid carcinoma to a lymph node in patients with a known history of thyroid carcinoma is often straightforward based on cytologic features. In challenging cases, IHC for TTF-1, calcitonin, and thyroglobulin may be useful in identifying the thyroid as a primary site for the nodal metastasis. Several studies have addressed the utilization of chemical assays for thyroglobulin on the FNA sample from neck lymph nodes [121,122]. Such an approach should be implemented with caution since clinical management of patients with benign or indeterminate lymph node FNAs containing detectable thyroglobulin remains undefined.

Ancillary studies that would permit re-classification of an indeterminate or suspicious thyroid FNAs into a benign or malignant category are highly desirable. Potential thyroid carcinoma-associated molecular markers include proteins (galectin-3, Cytokeratin-19, HBME-1), chromosomal translocations (*RET*/PTC, *PAX8*/*PPARG*), and genetic mutations (*BRAF*, *RAS*) [123-125]. This review has focused on molecular markers that have proven efficacy for the above stated indication. The specificity of several markers for thyroid carcinoma is promising, but based on current limited evidence, widespread clinical use will require further studies.

The indications for performing ancillary studies and the current state of the science for ancillary studies in thyroid FNA are summarized in Table 2.

**Table 2:** Review of Utilization of Ancillary Studies in Thyroid FNA

**Suspected medullary carcinoma**
• IHC panel (calcitonin, thyroglobulin, CEA, chromogranin)
• Serum calcitonin
**Suspected anaplastic carcinoma**
• IHC for pan-cytokeratin
**Suspected metastatic carcinoma**
• IHC for TTF-1 (If TTF-1 negative, then expand IHC panel based on cytomorphology and clinical setting to identify primary)
**Suspected lymphoma**
• Flow cytometric immunophenotyping
**Suspected parathyroid lesion**
• IHC for TTF-1, PTH, chromogranin
• May consider PTH level assessment on FNA sample
**Suspected metastatic thyroid carcinoma to lymph node**
• IHC for TTF-1, thyroglobulin, calcitonin
• May consider thyroglobulin level assessment on FNA sample
**Atypical/Borderline/Suspicious FNA**
• No specific tests based on insufficient evidence

### C. Sample preparation for each type of ancillary study

If IHC is anticipated, one dedicated pass should be performed for cell block preparation from an FNA sample. The routine use of thyroid core biopsy to perform IHC is not supported. If lymphoma is suspected, at least one dedicated pass in a supportive medium should be obtained for flow cytometric analysis. Ancillary studies to detect genetic alterations may require dedicated passes and special processing protocols depending on the analyte (DNA or RNA) and the methodology (FISH, PCR, RT-PCR).

## VI. Post FNA Options for Testing and Treatment

### A. Follow-up of "Non-diagnostic" FNA Results

A universally accepted approach to "Non-diagnostic" thyroid FNAs is currently lacking. The strategy reviewed here is based on the American Thyroid Association's proposal, recent literature and the NCI Conference on Thyroid FNA [9,48]. Non-diagnostic aspirates obtained from cystic and solid nodules are treated differently in follow-up strategies. Aspirates composed of pure colloid and lacking a cellular component are considered benign, but require close clinical and ultrasound follow-up. Aspirates of cysts containing blood and histiocytes but no epithelial component need correlation with ultrasound findings [19]. Many cystic nodules contain only colloid surrounded by a thin rim of benign epithelium. These cysts are at very low risk for harboring malignancy. Many authors recommend these cysts be managed by nonsurgical follow-up. Other authors point to the low, but real incidence of papillary carcinoma in cysts and recommend surgical resection after two "Non-diagnostic" aspirations. The timing of repeat needle aspiration has not been established, but 6 to 18 months appears reasonable.

Cystic lesions with a "Non-diagnostic" aspirate should undergo repeat FNA if ultrasound demonstrates suspicious areas [12,126]. The repeat FNA should be under ultrasound guidance and, when possible, intraprocedural review of the aspirated material by a cytopathologist is recommended [48,19]. When repeat FNA yields "Non-diagnostic" material, correlation with family history, close clinical and ultrasonographic follow-up is suggested [19].

Solid nodules with a "Non-diagnostic" aspirate should be re-aspirated with ultrasound guidance with, if possible, intraprocedural review by a cytopathologist. If repeat smears are "Non-diagnostic," surgery ought to be considered. If the patient is likely to return for follow-up and the nodule is 1 cm or less in size, close clinical follow-up with ultrasound examination is a reasonable alternative to surgery [19]. When growth of the nodule is detected during surveillance, excision is suggested but repeat FNA is an acceptable approach. In general, for both solid and cystic "Non-diagnostic" aspirates a waiting period of at least three months should elapse between the initial "Non-diagnostic" aspirate and reaspiration. If suspicion for carcinoma is high based on clinical or ultrasonographic findings, a shorter waiting period may be appropriate.

### B. Follow-up of "Benign" FNA Results

Management of patients with a "Benign" FNA diagnosis has varied between institutions. Because cytologically benign thyroid nodules are associated with up to 5% false negatives, these nodules require careful follow-up [16,127]. Patients with multiple thyroid nodules have the same risk of malignancy as those with a single nodule. Follow-up of patients with multiple nodules should be the same as those with a solitary nodule. Certain ultrasonographic characteristics (microcalcification, hypoechogenicity, intranodular hypervascularity) indicate a higher likelihood of malignancy [12,126]. Nodules with these features require more frequent clinical and ultrasonographic follow-up after a benign FNA. The false negative rate may be higher when FNAs are directed by palpation rather than by ultrasound [17,128,129]. Thus, palpation directed FNAs may require closer follow-up.

Medical suppressive therapy to confirm a benign cytologic diagnosis remains controversial. Multiple randomized trials have shown that thyroid hormone suppression may result in a decrease in nodule size in patient populations with borderline low iodine intake. The data are less convincing for populations ingesting sufficient iodine [130-132]. Rare examples of carcinomas suppressed by thyroid hormone have been reported. It is unclear that thyroid suppressive therapy is a reliable test for confirmation of a benign cytologic diagnosis of a solitary nodule.

Cytologically benign nodules can be followed clinically with interval ultrasound examination [19]. Benign nodules may be reaspirated or surgically removed when significant changes occur in their ultrasonographic appearance. Ultrasonography appears to be the best technique for detection of changes in nodule size[133]. There is no general agreement as to what constitutes a significant increase in nodule size. The American Thyroid Association (ATA) has suggested that a 20% increase in nodule diameter with a minimum increase in two or more dimensions of at least 2 mm is a reasonable definition for a significant change in nodule size [9]. The ATA has also recommended clinical follow-up of cytologically benign and easily palpable nodules occur at 6–18 month intervals. When nodules are not easily palpable, the recommendation is for serial ultrasound examinations at 6–18 month intervals [9].

Thyroid nodules cytologically diagnosed as benign require careful clinical follow-up. Easily palpable nodules may be followed clinically at 6–18 month intervals. Nodules which are not easily palpable should receive serial ultrasound examinations at 6–18 month intervals. The total duration of the follow-up period should be at least 3–5 years. When a 20% increase in nodule diameter or a minimum of 2 mm increase in two dimensions is detected, repeat FNA is performed. Repeat FNA should be performed if ultrasound abnormalities (irregular margins, central hypervascularization) develop. The repeat FNA should be under ultrasound guidance. Attendance of a pathologist at reaspiration is desirable. At this time, hormone suppressive therapy can not be recommended as a diagnostic maneuver for confirmation of benignity in a cytologically benign thyroid nodule. Ethanol ablation may be considered in selected patients.

### C. Follow-up of FNA specimens diagnosed as "Follicular lesion/Atypical/Borderline"

A variety of terms are employed to convey uncertainty about the significance of some thyroid cytologic findings. Such changes do not rise to the level of a significant concern for a follicular neoplasm meriting lobectomy (see section D), nor do they fit a "Suspicious for Malignancy" interpretation. Because of cytologic or architectural atypia, neither can such cases be reliably called benign. A variety of diagnostic headings are used for such cases including "Atypical Follicular Lesion", "Cellular Follicular Lesion" and "Indeterminate" [73,134]. The term "Atypical/Borderline" will be used for this discussion. Approximately 5–10% of the "Atypical/Borderline" category are malignant neoplasms. Given that this diagnostic category is associated with low specificity and a low positive predictive value, the appropriate follow-up for this category remains controversial. Some authorities have recommended repeat FNAs, repeat ultrasound scans, or repeat radio-nucleotide uptake studies. A repeat FNA is benign in about one-half of patients, obviating the need for surgery [71]. Radiological correlation may aid in improving the overall positive predictive value of this category. Besides increasing size, ultrasonographic features such as hypoechogenicity, irregular border, calcifications and abnormalities of vascularization all favor a malignant diagnosis [54,135].

Outside expert cytopathology consultation may be considered in cases with an "Atypical/Borderline" cytologic diagnosis. In general, a conservative approach may be taken. After a single "Atypical/Borderline' interpretation, a repeat FNA should be considered in 3 to 6 months. If the repeat FNA is "Atypical/Borderline" or worse, a surgical consultation should be considered [71].

### D. Follow-up of an FNA with the diagnosis of "Neoplasm (Follicular)"

This category has, in some reports, been termed "Suspicious for Follicular Neoplasm" [54,71]. The majority of cases in this category are adenomas, but 20 to 30% are carcinomas [73]. Patients with a diagnosis of "Follicular Neoplasm" should be referred for operative exploration. Usually a lobectomy is performed followed by histologic examination for capsular and vascular invasion.

The utility of frozen section evaluation for capsular or vascular invasion is controversial. Most participants at the NCI Meeting voiced the opinion that frozen section evaluation does not play a role in the separation of follicular adenoma from follicular carcinoma. Some surgeons may perform frozen section to determine the necessity for additional surgery, but there is no published data to support this practice. When frozen section is not utilized, initial surgery is lobectomy. If subsequent histologic examination discloses capsular or vascular invasion, the diagnosis of follicular carcinoma is made. Depending on the surgeon's discretion, histopathologic findings and clinical status of the patient reoperation with thyroidectomy may be performed.

### E. Follow-up of FNAs with a diagnosis of "Suspicious for Malignancy"

Approximately 50–75% of lesions cytologically diagnosed as "Suspicious for Malignancy" are papillary carcinomas [71,73,134]. Less commonly, other malignancies such as medullary carcinoma are included in this category. Patients with an FNA diagnosis of "Suspicious for Malignancy" should be referred for thyroid lobectomy. Subsequent operative intervention depends on histological review.

### F. Follow-up of "Malignant" FNA Results

This category refers to the histopathologic entities of papillary carcinoma, medullary carcinoma, lymphoma and anaplastic carcinoma. A comprehensive review of this subject concluded that the cytologic diagnosis of malignancy in a thyroid nodule should result in surgical consultation. Whenever possible, the type of carcinoma present should be stated in the cytology report. If a metastatic carcinoma is suspected, clinical and imaging studies should be undertaken to establish the primary site. At the surgeon's discretion, surgical intervention may initially be simple lobectomy or intraoperative frozen section examination to determine if total thyroidectomy should be performed. If frozen section is equivocal, the operative procedure is ended with lobectomy and further therapy is based on permanent sections. Depending on the surgeon's discretion and the characteristics of the malignancy, total thyroidectomy may be performed for a cytological diagnosis of papillary carcinoma. Controversy exists as to whether total thyroidectomy or unilateral lobectomy should be performed for papillary carcinoma. The selection of lobectomy versus thyroidectomy depends on the evaluation of the patient's clinical status and the size and nature of the papillary carcinoma. Total thyroidectomy may be accompanied by a central compartment dissection. For patients with large, bulky disease or recurrent laryngeal nerve dysfunction, preoperative cross sectional imaging should be considered as well as ultrasound imaging for lateral neck nodal disease.

## Competing interests

The author(s) declare that they have no competing interests.
